# Death of Eminent Medical Men

**Published:** 1849-10

**Authors:** 


DEATH OF EMINENT MEDICAL MEN.

The present year is remarkable for the mortality among the eminent professional men of our country. Some months ago it became our melancholy duty to notice the premature demise of Prof. Barbour, of St. Louis. Since that time the profession in every quarter of the land has been called upon to deplore the loss of valuable members. Among those who have been recently called away from the scenes of their labors and usefulness we have now to announce the names of Harrison, Brigham, and Butterfield.

The name of Professor Harrison, of Cincinnati, is familiar to the readers of the Journal. As an author we have had frequent occasions to speak of him, and he was equally well known as a learned and eloquent teacher in the medical schools of a neighboring city—first in the Cincinnati Medical College, and more recently in the Medical College of Ohio. At the time of his death he held the chair of Materia Medica and Therapeutics in the latter institution, having been transferred from the chair of Theory and Practice in the late reorganization of the Faculty. As a man and a teacher, Dr. Harrison was noted for his earnestness. His temperament was ardent, and he entered with the utmost zeal into whatever engaged his attention. No one could listen to him while speaking, whether in private circles or in public discussion, without a conviction that he felt himself to be the advocate of truth; and what he believed to be the truth he was ready, at all times and in all places, fearlessly to maintain. This earnestness of character, amounting to enthusiasm, his zeal for every measure tending to elevate his profession, his great industry, and the high moral tone which pervaded all his discourses, rendered Professor Harrison a valuable teacher, and his loss is one which the whole profession may well deplore. At the meeting of the Medical Association in Boston he was elected one of the vice presidents of that body, having at the previous meeting in Baltimore been appointed chairman of the committee on American Medical Literature, on which subject he read a report to the Association at its last meeting. He died in the prime of professional life, a victim to the epidemic which has swept away so many of his brethren. A more detailed biographical notice of the deceased is due to his elevated position and distinguished services, and will no doubt be prepared by his colleagues.

Dr. Brigham, who, at the time of his death, was Physician to the Asylum for the Insane, at Utica, N. Y., had won a wide celebrity by his writings on diseases of the mind, to which class of affections he had devoted the labors of his life. His authority is as often quoted by European writers as that of almost any other American author. The services which he has rendered to the unfortunate insane are very great, and he will have a place among those who have contributed to the remarkable progress of that department of practical medicine which our age has witnessed

Professor Butterfield, of Columbus, Ohio, was editor of the Ohio Medical & Surgical Journal, the publication of which was commenced at Columbus about a year since, and in the management of which, as has been well remarked by a cotemporary, "he exhibited a combination of the peculiar talents required for such an office." He died of consumption, under the pressure of which he wrote most of what appeared in his journal from his pen, "*hoping*," as he expressed it, "all the while for better times." Dr. Butterfield was formerly of Lowell, Massachusetts, but from the origin of the institution occupied the chair of Practice of Medicine in the Starling Medical College, at Columbus.

We will not close these notices without allusion to a name, unknown indeed to fame, but highly honored in the sphere where his useful labors were performed. Dr. James M. Jetton, of Murfreesborough, Tennessee, died of cholera early in August last, in the neighboring town of Lebanon. On hearing that one of the physicians of that afflicted town had perished by the epidemic and that nearly all the rest were disabled by it, in consequence of which the citizens were suffering for the want of medical aid, he at once quitted his home and practice and, with his wife, repaired to the scene of suffering and danger. In two days after he reached the infected spot his wife sickened with the pestilence and died. In a short time he was himself seized with the disease, which, owing, in part perhaps, to the lack of proper professional care, proved fatal in a few hours. Dr. Jetton had prepared himself carefully for the duties of medical practice, and, had he been permitted to pass safely through the epidemic to which he exposed himself, would have attained in a short time to an enviable rank in his profession.

